# Pediatric liver and kidney transplantation in the era of COVID-19: a follow-up study from a tertiary referral center in Iran

**DOI:** 10.1186/s12893-021-01226-y

**Published:** 2021-05-11

**Authors:** Mojtaba Shafiekhani, Kourosh Kazemi, Ali Bahador, Mohammad Hadi Imanieh, Parisa Karimzadeh

**Affiliations:** 1 Shiraz Transplant Research Center, Shiraz University of Medical Sciences, Shiraz, Iran; 2 Shiraz Transplant Center, Abu-Ali Sina Hospital, Shiraz University of Medical Sciences, Shiraz, Iran; 3 Department of Clinical Pharmacy, Faculty of Pharmacy, Shiraz University of Medical Sciences, Shiraz, Iran; 4 Department of Pediatrics Gastroenterology and Hepatology, Shiraz University of Medical Sciences, Shiraz, Iran

**Keywords:** COVID-19, Liver transplantation, Kidney transplantation, Pediatrics

## Abstract

**Background:**

We aimed to evaluate the impact of COVID-19 pandemic on pediatric transplant outcomes and determine whether to continue pediatric transplant activity or not, and how policies intended our center has been effective in preventing COVID-19 among organ transplant recipients.

**Methods:**

We conducted a single-center, retrospective, cohort study of hospitalized pediatrics after organ transplantation at Shiraz transplant center since March to August 2020. All liver and kidney transplanted children were included the study and their laboratory and clinical related COVID-19 characteristics were followed up till 3 months after transplantation during hospitalization period and then weekly by the transplant committee.

**Results:**

Fifty-one patients underwent transplantation including 11 kidney and 40 liver recipients. The mean age of the pediatric cases was 6.72 ± 5.47 years. A total of 11 patients died due to post-transplant complications, while none of the patients presented any sign or symptoms in favor of COVID-19 in the hospital course after transplantation. Six transplants including 2 kidney and 4 liver were canceled when positive PCR tests were detected in their donors before the surgery. In the 3 months of follow up, two patients presented with symptoms including high grade fever, malaise, rhinorrhea, and GI symptoms. Both patients had two negative PCR, and no radiologic or laboratory results regarding COVID-19 were also detected. One had positive influenza PCR, while the second one had a positive serologic test for EBV; CT, computed tomography

**Conclusion:**

Transplant programs could continue their activities during the COVID-19 pandemic with specific case selection, accurate screening methods and following protective protocols.

**Supplementary Information:**

The online version contains supplementary material available at 10.1186/s12893-021-01226-y.

## Introduction

The global pandemic of the severe acute respiratory coronavirus 2 (SARS-CoV-2) has emerged as a life-threatening infection, declared as a global pandemic by the Center for World Health Organization (WHO) on March 11 [1]. According to WHO data, there have been reports of a total of 35,109,317 confirmed cases of COVID-19, including 1,035,341 deaths worldwide, on 5 October 2020 (3:54 pm CEST); of them, 471,772 cases and 26975 deaths were registered in Iran [2].

Although pediatric data regarding COVID-19 is limited, published studies suggest a less than 5% involvement of children (< 18 years-old) with a less severe manifestations in comparison to adults [3, 4]. However, severe outcomes have been reported in children including hospitalization, ICU admission, and death [5]. Moreover, children with underlying conditions such as chronic lung disease (including asthma), cardiovascular disease, and immunosuppression are at a higher risk of severity of the disease [3].

Solid organ transplant recipients, normally maintained in an immunosuppressed state to avoid organ rejection, are expected to be more susceptible for COVID-19 due to their immune status [6]. Besides, they might have poorer outcomes in the case of COVID-19 due to their comorbidities, while their immunosuppressive management is also of a great concern [7, 8]. Therefore, dealing with this situation with respect to issues around donors or recipients, and also approaches regarding new transplant should be aligned with international recommendations.

The aim of the present study was to evaluate the impact of COVID-19 pandemic on pediatric transplant operations, whether to continue pediatric transplant activity or not, and transplant recipients diagnosed with COVID-19 among liver and kidney transplanted children who referred to our center as the only tertiary referral hospital in Iran during March 27, 2020 and August 27, 2020 based on the clinical and laboratory data.

## Patients and methods

### Study design

This retrospective study was done from March to August 2020 in Shiraz transplant center, which is affiliated to Shiraz University of Medical Sciences as a tertiary referral center for pediatric transplantation in Shiraz, Iran. All liver and kidney transplanted patients under 18 years old were included the study and their laboratory and clinical characteristics were followed up till 3 months after their transplant weekly by the transplant committee. This study was approved by the ethics committee of Shiraz University of Medical Sciences and all methods were also performed in accordance with the relevant guidelines and regulations under the committee supervision. Moreover, written informed consent was obtained from all the patients' parents or guardians.

### Data collection

Medical and laboratory records from transplantation till 3 months later were extracted from available electronic inpatient medical record databases and reviewed to obtain demographic (sex.age, PELD score, type of transplantation and donors, causes of transplantations), clinical (sign and symptoms regarding COVID-19, length of ICU and hospital stay, rejection and re-hospitalization episodes and live or death situation), laboratory(hematologic and biochemistry indexes regarding graft function, plasma prograf and cyclosporin level, inflammatory indexes such as ESR,CRP) and therapeutic information(anti-viral therapy and immunosuppressive regimen) as well as post-transplant complications and outcomes.

### Pre transplant evaluation policies

In the beginning, all transplant candidates and donors were accurately evaluated by Pediatric Infectious Diseases (ID) specialist regarding COVID-19 symptoms. A normal spiral chest computed tomography (CT) as well as two negative nasopharyngeal swabs for SARS-CoV-2 PCR with 48-h interval was required from both living donor and recipients before planned hospitalizations or procedures. However, one negative nasopharyngeal swab for SARS-CoV-2 PCR without any suggestive COVID-19 symptoms is enough from deceased donor or in case of emergency. A confirmed case of COVID-19 was defined by a positive laboratory testing of SARS-CoV-2 by reverse transcription polymerase chain reaction (RT-PCR) from a nasopharyngeal sample as follow.

Nasopharyngeal swab was collected from single nostril and specimens were collected and stored in a collection tube with 5 mL virus preservation solution. RNA was isolated with automatic nucleic acid extraction system. The RT-PCR assay detecting both nucleocapsid protein (N) and open reading frame 1ab (ORF1ab) genes simultaneously.RT-PCR assay was performed with 96E real-time PCR system in a volume of 25 μL using the following conditions: 50 °C for 15 min, 95 °C for 15 min, 45 cycles of 94 °C for 15 s, 55 °C for 45 s for fluorescence collection. The cutoff cycle threshold (Ct) value was 40 for both genes, and the Ct values of both genes were less than 40 was defined as positive [9].

### Specific COVID-19 management (COVID-19 protocol)

All transplanted patients were visited daily by a transplant surgeon, pediatric gastroenterohepatologist or pediatric nephrologists and a Pediatric ID specialist after transplantation till hospitalization period and routinely evaluated for any clinical signs or symptoms suggestive of COVID-19 such as cough, fever, myalgia, dyspnea, diarrhea, nausea and vomiting, unexplained hematological abnormalities, elevated inflammatory markers and any others related symptoms. If an infection was suspected, alongside with SARS-CoV-2 PCR, laboratory tests including complete blood count (CBC), Erythrocyte Sedimentation Rate (ESR), baseline serum C-reactive protein (CRP), lactate dehydrogenase (LDH), ferritin, D-dimer, creatine phosphokinase (CPK), and procalcitonin were sent. In the confirmed case of COVID-19 diseases, management and immunosuppressive changes were done according to published scientific guidelines [10–12].

### Follow-ups

Outpatient visits were performed both in person in the clinic weekly by the transplant surgeon, pediatric gastroenterohepatologist or pediatric nephrologists, while facial surgical masks were worn by health-care professionals, patients, and parents at all time and telephone pre-triage follow-ups were also done as remote patient monitoring, asking about suggestive symptoms of COVID-19 infection including ongoing fever, gastrointestinal vs. respiratory symptoms in the patient or in the caregiver. This telephone pre-triage was continued until 3 months after their transplantation and extended to the hospital, if needed.

### Statistical analysis

Categorical variables were described as frequency rates and percentages, and continuous variables were described using mean, median, and interquartile range (IQR) values. Means for continuous variables were compared using independent group t-tests when the data were normally distributed. All statistical analyses were performed using SPSS (Statistical Package for the Social Sciences) version 20.0 software (SPSS Inc). A p value of < 0.0 5 was regarded as statistically significant.

## Results

Fifty-one patients underwent transplantation including 11 kidney and 40 liver recipients during the study period from March 27, 2020 and August 27, 2020. According to the global impact of COVID-19 on our center practices, the number of liver and kidney transplantations had decreased by 25.8% and 32.1% compared to the same period last year, respectively. The demographic data are included in Table 1. As shown, the mean age of the pediatric cases was 6.72 ± 5.47 years (range, 12 to 18 years). In total, 33(64.7%) patients were female in comparison to 18 (35.3%) male patients.

**Table 1:** Demographic data of pediatric cases who had undergone liver and kidney transplantation (N = 51)

Variable	Total (N or Mean ± SD)	Kidney Tx recipients (n = 11)	Liver Tx recipients (n = 40)
Age (years)	6.72 ± 5.47	7.72 ± 4.91	6.11 ± 5.80
Sex			
Male	33 (64.7)	4	29
Female	18 (35.3)	7	11
White blood cells	8.79 ± 5.57	9.01 ± 6.77	8.66 ± 5.32
Creatinine *(mg/dL)*	0.64 ± 1.04	0.80 ± 1.00	0.61 ± 0.95
Aspartate transaminase (U/L)	161.58 ± 132.52	21.39 ± 19.88	171.21 ± 134.01
Alanine aminotransferase (U/L)	98.75 ± 80.93	14.33 ± 10.00	112.67 ± 99.07
Total Bilirubin (mg/dL)	17.96 ± 16.88	1.22 ± 1.00	19.08 ± 17.23
PELD score	21.20 ± 8.88	–	21.20 ± 8.88
Patients outcome			
Alive	40 (78.4)	11	29
Expired	11 (21.6)	0	11
Immunosuppressive regimen			
Tacrolimus + prednisolone	32(62.74%)	1	31
Mycophenolate mofetil + tacrolimus	9 (17.64%)	1	8
Cyclosporine + prednisolone	10(19.60%)	9	1
Induction regimen			
Methylprednisolone	41(80.39%)	8	33
*Thymoglobulin*	10 (19.6%)	3	7
Post-transplant laboratory value			
O_2_ saturation (%)	91	93	90
Lymphocytopenia	5 (10.6%)	2	3
Lymphocytosis	13 (27.7%)	1	12
Elevated CRP	19 (51.4%)	10	9
Elevated ESR	8 (22.9%)	3	5
Prograf level(ng/dl)	4.91 ± 2.21	5.82 ± 1.99	4.88 ± 2.01
Cyclosporine level	190.34 ± 34.12	194.00 ± 36.21	188.30 ± 30.01
CMV PCR positive	4 (14.8%)	3	1

Tx, transplant; PELD, Pediatric end-stage liver disease; CRP, C-reactive protein; ESR, erythrocyte sedimentation rate; CMV, *cytomegalovirus*; PCR, polymerase chain reaction

Regarding the underlying disease presented in Table 1, among liver transplant patients, Biliary atresia accounted for the majority of cases, followed by Progressive familial intrahepatic cholestasis (PFIC), Wilson, while Focal segmental glomerulosclerosis (FSGS) was the main cause of kidney transplant among our patients. Among liver transplant recipients, the median Pediatric end-stage liver disease (PELD) score was 21.20 ± 8.8 (6–49). The baseline laboratory results are detailed in Additional file 1: Table S1. Twenty-four transplantations (47.1%) were from living-donor and 27(52.9%) were from deceased -donor.

At the end of the study, 40 (78.4%) patients were discharged with median ICU admission length of 27.97 days (13–77 days). Eleven patients died after liver transplant due to post-transplant complications including bleeding [1], portal vein thrombosis (PVT) [1], Disseminated intravascular coagulation (DIC) [4], and graft failure [5], whereas all kidney transplant patients were alive.

None of the patients presented any sign or symptoms in favor of COVID-19 in the length of hospital stay. Six transplants including 2 kidney (both from deceased donors) and 4 liver (all of them were living donors) were canceled when positive PCR tests were detected in their donors before the surgery.

Among these patients, 41 (80%) were not readmitted after discharge from the hospital in the 3 months of our follow up. Among other 10 patients, two were admitted due to ascites, four due to elevated liver enzyme and creatinine level, and four due to bacterial infections.

In the 3 months of follow up, two patients presented with symptoms including high grade fever, malaise, rhinorrhea, and GI symptoms and were evaluated with suspicion to COVID-19 by pediatric ID specialist. Both patients had two negative PCR, and no radiologic or laboratory results such as lymphopenia or decrease in O2 saturation level were detected. One of them had positive influenza PCR test and were treated with Oseltamivir, while the second one had a positive serologic test for EBV which was treated by Acetaminophen and reduction in immunosuppressive regimen.

## Discussion

As immunosuppression and comorbidities predominantly afflict the solid organ transplant recipients, especially pediatric groups, COVID-19 is a matter of concern for many pediatric transplantation centers. At the moment, little is known about the exact impact of COVID-19 in solid transplant patient outcome. In our study, we retrospectively reviewed 51 pediatric transplanted patients after their surgery with a 3 month follow up. Our findings showed a slight reduction in the rate of transplantation in our center compared to last year as well as no COVID-19 involvement in the new organ recipients.

From the pediatric perspective, it could be assumed that children have a less severe disease in comparison to adults, and they corresponded with only 5% of the total number of patients involved with COVID-19 in China [13]. While this could not be related to transmission chance since viral transmission in children is mentioned to be apparently similar to adult group in an epidemiological analysis by Qifang et al. of 391 cases and their 1286 close contacts [13].

Some hypotheses may explain the benign course of COVID-19 in pediatric cases compared with adults. First, viral clearance is more rapid in children which may lead to inflammatory response reduction which appears to be particularly important drivers of tissue damage during infection [14, 15]. Second, children do not carry degenerative features of aging that is considered as COVID-19 risk factor [14, 15]. Milder pattern of the disease may also be related to the lower maturity and the binding capacity of the angiotensin converting enzyme II, probably the virus cell receptor [14].

Immunocompromised patients’ reports are also few; this could illustrate their lower chance of involvement with the virus. Although earliest report of liver transplant children was available in Bergamo [16], their negligible symptoms suggested that the immunological impairment itself may be the cause of blunting the inflammatory cascades and cytokine release since the inflammatory reaction enhances the damage of the disease [6]. Another case–control study by Chaudhry and his colleague on the clinical characteristics and outcomes of COVID-19 in solid organ transplant recipients associated the increasing age and clinical severity with mortality. While transplant status itself was not considered as a contributing factor with mortality [6]. Another study demonstrated that Tacrolimus, widely used in transplant patients, strongly inhibited the growth of human coronavirus SARS-CoV, too [17]. Furthermore, tight social distancing which is usually experienced by immunocompromised patients is considered as the main factor in their lower infection risks [18]. Our preliminary experience, in agreement with recent data from of China and Italy which showed that none of the patients was followed for transplantation or autoimmune liver disease, developed a clinical pulmonary disease, except for some positive tests for SARSCoV-2. They assert that immunosuppressed patients are not at a higher risk of severe complications in comparison to the general population, both in children and adults [19].

Pediatric transplantation (0–17 years old) is a highly complex procedure that makes coordination of resources and specialized professionals of a great importance [20]. A total rate of 108 pediatric transplant per year reported an activity of approximately 317 pediatric transplants from 2017 to 2019, while a 29.2% reduction was seen in this period in our center, which is compatible with the result of European centers that show COVID-19 pandemic substantial negative effect on pediatric transplantation activity as well as outpatient visits due to the fear of SARS-CoV-2 transmission risk, and shortage of hospital bed capacity and staff. On the other hand, it is noteworthy to mention the liver transplantation center of Hong Kong which shifted its resources to SARS patients during the SARS pandemic and performed no transplants for 6 months [21]. This approach directly affected the quality of care in transplanted patients and liver transplant candidates though no case of severe pneumonia was also recorded [21]. The long-term continuation of these limitations surely has dire consequences on pediatric transplant recipients as well as children on the transplant waiting list, reducing their access to close monitoring and follow ups. Therefore, optimizing the resource in specialized transplant center and establishing guidelines in terms of pediatric transplant recipients and candidates during the COVID-19 pandemic are required. D’antiga also suggested that coronaviruses have not been shown to cause a more severe disease in immunosuppressed patients, so that there is no reason to postpone lifesaving treatments, such as transplantation, both in children and in adults during coronavirus pandemics [16].

However, as the only pediatric transplant center in the country, our centers had to remain active during the period and the living donor was reserved for urgent cases, while deceased donors were available with specific adaptations of the protocols for COVID-19 screening, although in both cases the accessibility was notably reduced. For complex patients, prioritization of which patients in the list for transplant in the period is regarded to Higher PELD score and acute liver failures. Besides, outpatient activities are continued both in person, and through telemedicine. In our protocol, a normal spiral chest CT as well as two negative nasopharyngeal swabs for SARS-CoV-2 with 48-h interval were taken from both living donors and recipients before planned hospitalizations or procedures which exclude 6 donors from the list due to their positive PCR test.

There is no final opinion regarding pre-transplant evaluation. Some centers use PCR and some Antibody test, while others only rely on spiral chest CT [22, 23]. The timing of tests varies from 1 week to 1 day before transplant in different studies [24]. In our study, the donor was excluded from transplant list if he/she presented with a positive PCR test, while other centers checked if PCR became negative or Antibody was presented [25].

This study has limitations. Since we are the only pediatric transplant center in Iran, our results cannot be compared with other less equipped centers to generalize. Even though our median follow-up was 3 months, long-term follow up needs further studies.

## Conclusion

In conclusion, despite the greater proportion of coexisting conditions and immune suppression, we concluded that solid organ transplant recipients seem not to have greater risk of COVID-19 involvement according our results but long term evaluation as well as multi-center data is also needed for further determination. Therefore, transplant programs could continue their activities during the COVID-19 pandemic with specific case selection, accurate screening methods while following protective protocols.

## Supplementary Information

**Additional file 1: Table S1.** Demographic data of pediatric cases who had undergone liver and kidney transplantation (N=51).

## Data Availability

The datasets generated and/or analysed during the current study are not publicly available due their containing information that could compromise the privacy of research participants but are available from the corresponding author on reasonable request.

## Abbreviations

CBC: Complete blood count
CMV: Cytomegalovirus
COVID-19: Coronavirus disease 2019
CPK: Creatine phosphokinase
CRP: C-reactive protein
DIC: Disseminated intravascular coagulation
EBV: Epstein Barr virus
ESR: Erythrocyte Sedimentation Rate
FSGS: Focal segmental glomerulosclerosis
GI: Gastrointestinal
ICU: Intensive care unit
ID: Infectious Diseases
LDH: Lactate dehydrogenase
PCR: Polymerase chain reaction
PELD: Pediatric end-stage liver disease
PFIC: Progressive familial intrahepatic cholestasis
PVT: Portal vein thrombosis
RT-PCR: Reverse transcription polymerase chain reaction
SARS-CoV-2: Severe acute respiratory coronavirus 2
WHO: World Health Organization
