# Supplementary material for: Pediatric liver and kidney transplantation in the era of COVID-19: a follow-up study from a tertiary referral center in Iran
Source: BMC Surg. 2021 May 11;21:240. doi: 10.1186/s12893-021-01226-y (PMC8112472; doi:10.1186/s12893-021-01226-y)
Supplement: Supplementary file 1 — Additional file 1: Table S1. Demographic data of pediatric cases who had undergone liver and kidney transplantation (N=51). [file 12893_2021_1226_MOESM1_ESM.docx]

**Table S1. Demographic data of pediatric cases who had undergone liver and kidney transplantation (N=51)**

| **Variable** | **Total (N or Mean±SD)** | **Kidney Tx recipients (n=11)** | **Liver Tx recipients**  **(n=40)** |
| --- | --- | --- | --- |
| **Age (years)** | 6.72 ± 5.47 | 7.72±4.91 | 6.11±5.80 |
| **Sex** | |  |  |
| Male | 33 (64.7) | 4 | 29 |
| Female | 18 (35.3) | 7 | 11 |
| **White blood cells** | 8.79±5.57 | 9.01±6.77 | 8.66±5.32 |
| **Creatinine** *(mg/dL)* | 0.64±1.04 | 0.80±1.00 | 0.61±0.95 |
| **Aspartate transaminase (U/L)** | 161.58±132.52 | 21.39±19.88 | 171.21±134.01 |
| **Alanine aminotransferase (U/L)** | 98.75±80.93 | 14.33±10.00 | 112.67±99.07 |
| **Total Bilirubin (mg/dL)** | 17.96±16.88 | 1.22±1.00 | 19.08±17.23 |
| **PELD score** | 21.20 ± 8.88 | - | 21.20 ± 8.88 |
| **Patients outcome** | |  |  |
| Alive | 40 (78.4) | 11 | 29 |
| Expired | 11 (21.6) | 0 | 11 |
| **Immunosuppressive regimen** | |  |  |
| Tacrolimus +prednisolone | 32(62.74%) (32.3%) | 1 | 31 |
| mycophenolate mofetil +tacrolimus | 9 (17.64%) | 1 | 8 |
| Cyclosporine +prednisolone | 10(19.60%) | 9 | 1 |
| **Induction regimen** |  |  |  |
| Methylprednisolone | 41(80.39%) | 8 | 33 |
| Thymoglobulin | 10 (19.6%) | 3 | 7 |
| **Post-transplant laboratory value)** |  |  |  |
| **O_2_ saturation (%)** | 91 | 93 | 90 |
| Lymphocytopenia | 5 (10.6%) | 2 | 3 |
| Lymphocytosis | 13 (27.7%) | 1 | 12 |
| Elevated CRP | 19 (51.4%) | 10 | 9 |
| Elevated ESR | 8 (22.9%) | 3 | 5 |
| Prograf level(ng/dl) | 4.91±2.21 | 5.82±1.99 | 4.88±2.01 |
| Cyclosporine level | 190.34±34.12 | 194.00±36.21 | 188.30±30.01 |
| CMV PCR positive | 4 (14.8%) | 3 | 1 |

Tx: transplant, PELD: Pediatric end-stage liver disease, CRP: C-reactive protein, ESR: erythrocyte sedimentation rate CMV: cytomegalovirus, PCR, polymerase chain reaction
